# The changing pattern of common respiratory viruses among children from 2018 to 2021 in Wuhan, China

**DOI:** 10.1007/s00705-023-05891-7

**Published:** 2023-11-14

**Authors:** Lu Wan, Liangyu Li, Haiyue Zhang, Chan Liu, Ruiyun Li, Xiaojun Wu, Jianjun Chen

**Affiliations:** 1 Department of Pulmonary and Critical Care Medicine, Renmin Hospital of Wuhan University, Wuhan, Hubei China; 2 Present Address: CAS Key Laboratory of Special Pathogens, Center for Biosafety Mega Science, Wuhan Institute of Virology, Chinese Academy of Sciences, Wuhan, Hubei China

**Keywords:** Respiratory virus, Epidemiologic features, Non-pharmaceutical interventions, Children, COVID-19

## Abstract

**Background:**

Acute respiratory infections in children are a global public health challenge. Owing to the coronavirus disease (COVID-19) pandemic, non-pharmaceutical interventions, including patient isolation, social distancing, hand washing, and mask wearing, have been widely implemented, impacting the transmission of common respiratory viruses. The aim of this study was to clarify the epidemiological features of respiratory viruses in children less than 14 years of age in Wuhan before and after COVID-19.

**Methods:**

Respiratory specimens were collected from patients aged < 14 years at two hospitals in Wuhan, China, from January 2018 to December 2021. Seven respiratory viruses were identified using an immunofluorescence assay. Pathogen profiles and seasonality were analysed.

**Results:**

The number of visits and virus detection rate decreased dramatically after February 2020. The respiratory virus detection rate peaked in January and December and decreased dramatically in February and August. The detection rate was lower in 2021 than in 2018 and 2019. Respiratory syncytial virus (RSV) was identified as the leading pathogen in children aged < 1 year and 1–4 years before and after the COVID-19 pandemic. In children aged 5–14 years, influenza virus was detected at the highest rate before, and RSV after, the COVID-19 pandemic. RSV was the most common virus in coinfections.

**Conclusions:**

This study revealed the epidemiological patterns of common respiratory viruses from 2018 to 2021. The spectrum of pathogens involved in paediatric respiratory infections had partly changed. Non-pharmaceutical interventions resulted in fewer opportunities for the spread of common viruses but also in an “immunity debt” that could have negative consequences when the pandemic is under control in Wuhan.

**Supplementary Information:**

The online version contains supplementary material available at 10.1007/s00705-023-05891-7.

Acute respiratory infections (ARIs) are responsible for high paediatric mortality and morbidity rates, posing a substantial threat to human health worldwide [1]. Respiratory viruses are the predominant pathogens that cause ARIs. Respiratory diseases are prevalent in infants and children aged < 5 years [2–5]. In a meta-analysis, it was estimated that 33 million RSV-associated acute lower respiratory infections occurred globally in children aged 0–60 months in 2019, with one in five episodes occurring in infants aged 0–6 months [6], and the proportion of patients admitted to a hospital for acute respiratory infection who were positive for RSV was highest in high-income countries for children aged 0–60 months [6]. The Chinese government has taken effective measures to treat patients with coronavirus disease (COVID-19) and implemented non-pharmaceutical interventions to prevent viral transmission; however, many children were still diagnosed with viral respiratory infections throughout the year [7]. Therefore, epidemiological surveillance of respiratory viruses in children is essential. Parainfluenza virus (PIV), respiratory syncytial virus (RSV), adenovirus (ADV), and influenza virus are common viruses that cause paediatric respiratory tract infections [8–11], especially in kindergartens, schools, and other high-density settings. Children with suspected ARI underwent evaluation for seven different respiratory viruses.

The ongoing COVID-19 pandemic is a public health emergency of international concern; it was first reported at the end of 2019 [12–15]. Consequently, residents of Wuhan were confined from 23 January to 8 April 2020. Meanwhile, the government had adopted both pharmaceutical and non-pharmaceutical interventions in the fight against the pandemic. Non-pharmaceutical interventions (NPIs), including confinement, social distancing, hand washing, and mask wearing, were implemented to prevent or slow down the spread of COVID-19 around the world. Numerous studies have indicated that the ARI rate in the paediatric population decreased after the COVID-19 pandemic [16–21]. In the short term, this decrease was welcome, as it prevented an additional overload of hospital wards and intensive care units during the COVID-19 epidemic. However, infections with these common respiratory viruses typically occur during early childhood and are almost unavoidable in the first years. A lack of immune stimulation due to NPIs causes an“immunity debt”that could have negative consequences when the pandemic is under control. Mathematical models suggest that RSV, and possibly influenza, epidemics may be more intense in the coming years [22]. Global surveillance of viral respiratory infections should continue over the long term. Epidemiological investigations, especially of disease aetiology, remain important for guiding paediatricians in the diagnosis and treatment of respiratory infectious diseases in children. Here, we investigated the epidemiological features of respiratory viruses by conducting a study on viral respiratory pathogens, seasonality, and clinical characteristics of respiratory diseases in children in Wuhan from 2018 to 2021.

## Materials and methods

### Patient enrollment

This retrospective epidemiological study investigated common respiratory viruses in patients under 14 years of age at two hospitals in Wuhan from January 2018 to December 2021. The inclusion criterion was the presence of at least one of the following symptoms/signs: fever, cough, chills, expectoration, nasal congestion, sore throat, chest pain, tachypnoea, or abnormal breath sounds. Children with COVID-19 were excluded from the study for observing the trends of the common respiratory viruses. A total of 54,171 patients with suspected ARI underwent evaluation for seven different respiratory viruses that are prevalent in China. Both outpatients and inpatients were recruited.

### Laboratory diagnosis

Throat, nasal, or nasopharyngeal swabs were obtained from children with suspected ARI. Specimens were placed into collection tubes with virus preservation solution and stored at 2–8 ℃ for 48 h until they were tested. Samples were tested for influenza A and B virus, RSV, ADV, PIV1, PIV2, and PIV3 as part of the national surveillance program using an immunofluorescence assay. Procedure-specific dosage and time recommendations were strictly followed [23]. The commercial kit used for detection was D^3^ Ultra DFA Respiratory Virus Screening and ID Kit (Diagnostic Hybrids, Inc, Athens, Ohio, USA).

### Groups

The enrolled patients were divided into three groups according to age: <1 year, 1–4 years, and 5–14 years. Positive patients were divided into single virus infection and coinfection groups.

### Data management and statistical analysis

All of the data were extracted from electronic medical records (Shanghai Ruimei Laboratory Information System) from 2018 to 2021. Descriptive statistics included categorical variables presented as frequencies and continuous variables presented as medians and interquartile ranges (IQRs). Pearson’s chi-square test or Fisher’s exact test was performed to compare categorical variables between groups. All statistical analysis was performed using IBM SPSS Statistics, Version 26.0 (IBM Corp., Armonk, NY), and interrelationships between variables were assessed at a significance level of *P* < 0.05.

### Ethical approval

The procedures followed in this study were in accordance with the principles of the Declaration of Helsinki (1964, amended most recently in 2008) of the World Medical Association. The protocol was approved by the Ethics Commission of Renmin Hospital of Wuhan University. Written informed consent was obtained from a parent and/or legal guardian for study participation for minors. This work was supported by the National Natural Science Foundation of China (81961138013, 31970174) and the Key Program of Chinese Academy of Sciences (CAS) (KJZD-SW-L11).

## Results

### Study population

**Fig. 1:**
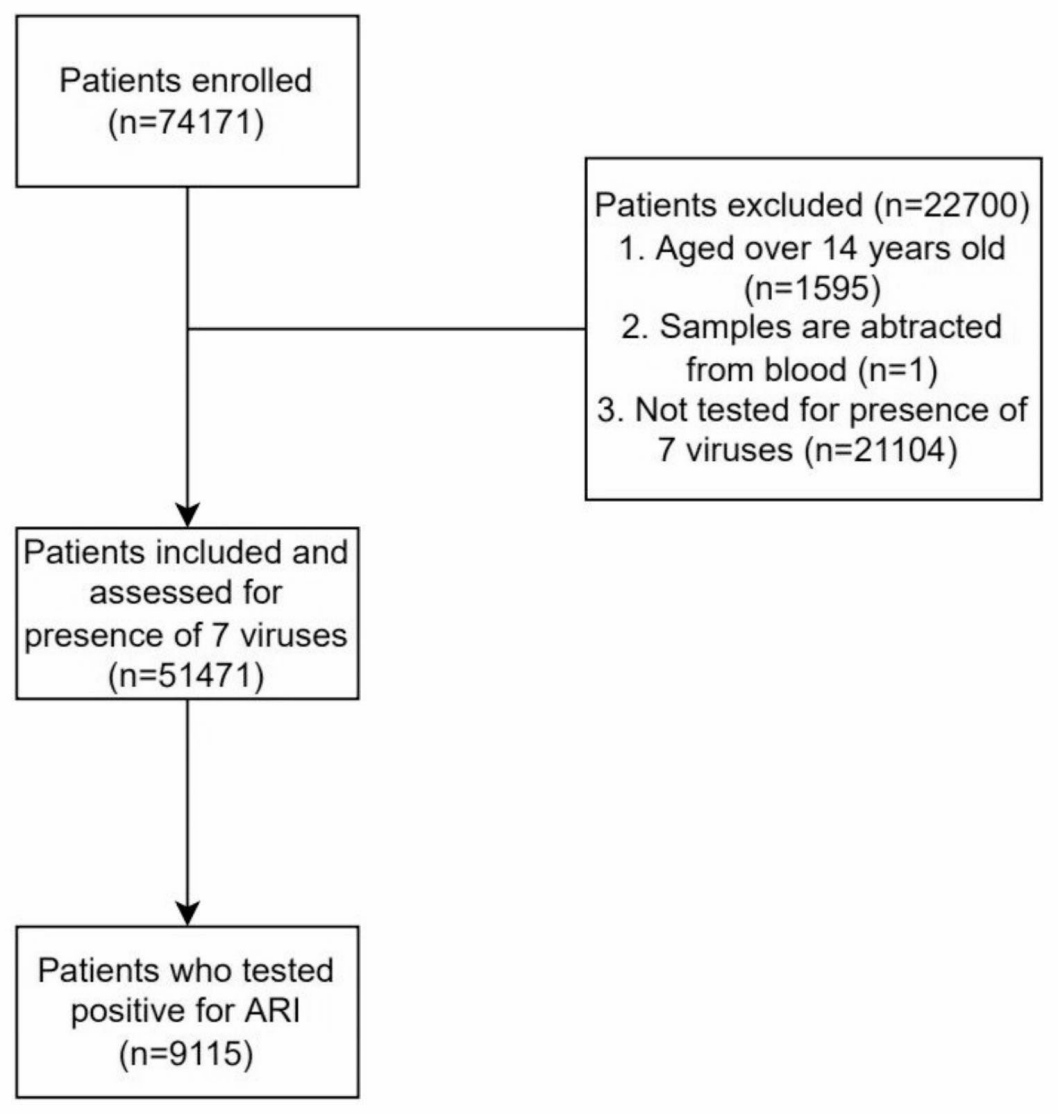
Study flowchart

**Fig. 2:**
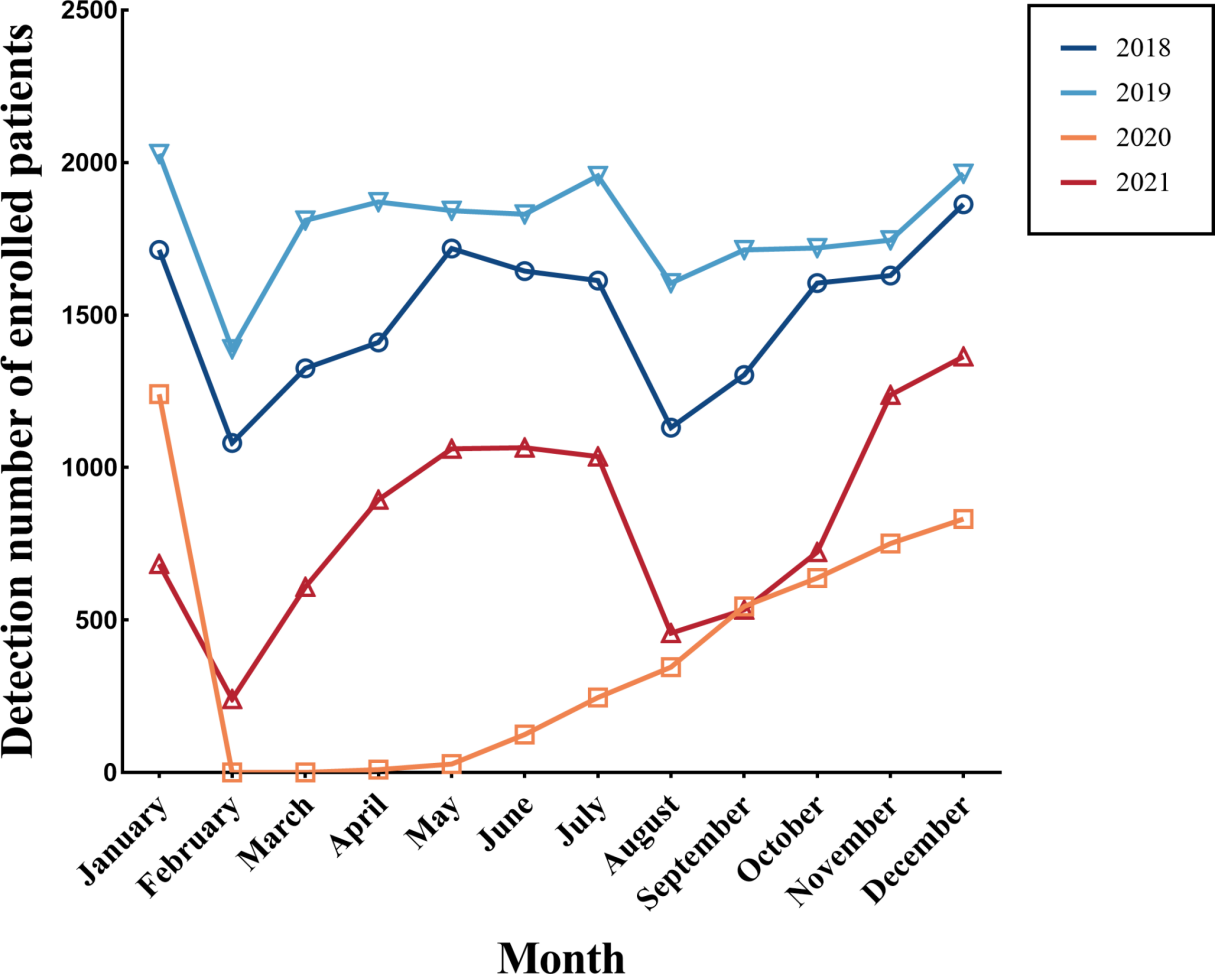
Monthly distribution of enrolled children (< 14 years) from 2018 to 2021. The number of patients with ARIs peaked in January and December and was lowest in February and August. The number of patients increased from February to June; thereafter, it decreased gradually until August. In Wuhan, the spring festival is in February, and summer vacation is in August. There was a brief outbreak in August 2021

Data from 54,171 children from Renmin Hospital of Wuhan University (two districts, 20 km apart) in Wuhan who underwent testing for seven different respiratory viruses from 2018 to 2021 were available for the final analysis. The study flowchart is shown in Fig. 1. The median age of the patients was 4 years (IQR 2–5 years, 77.8% were aged < 5 years). There were 32,000 boys (59.07%) and 22,171 girls (40.93%) enrolled in the study (Table 1). The seasonal variation of enrolled patients was similar in 2018, 2019, and 2021 (Fig. 2, Supplementary Table S1).

**Table 1:** Demographics of the enrolled patients (< 14 years)

Category	2018	2019	2020	2021	Total
	n = 18,035, no. (%)	n = 21,471, no. (%)	n = 4760, no. (%)	n = 9905, no. (%)	n = 54,171, no. (%)
Boys	10 781 (59.78)	12 569 (58.54)	2869 (60.27)	5781 (58.36)	32,000 (59.07)
Girls	7254 (40.22)	8902 (49.36)	1891 (39.73)	4124 (41.64)	22,171 (40.93)
Age					
< 1 year	3538 (19.62)	4156 (19.36)	926 (19.45)	1518 (15.33)	10,138 (18.71)
1–4 years	10,805 (59.91)	12,384 (57.68)	2748 (57.73)	6086 (61.44)	32,023 (59.11)
5–14 years	3692 (20.47)	4931 (22.97)	1086 (22.82)	2301 (23.23)	12,010 (22.17)

There was no significance difference in the number of patients with ARIs among the three age groups (*P* = 0.116)

### Respiratory virus infection rate

**Fig. 3:**
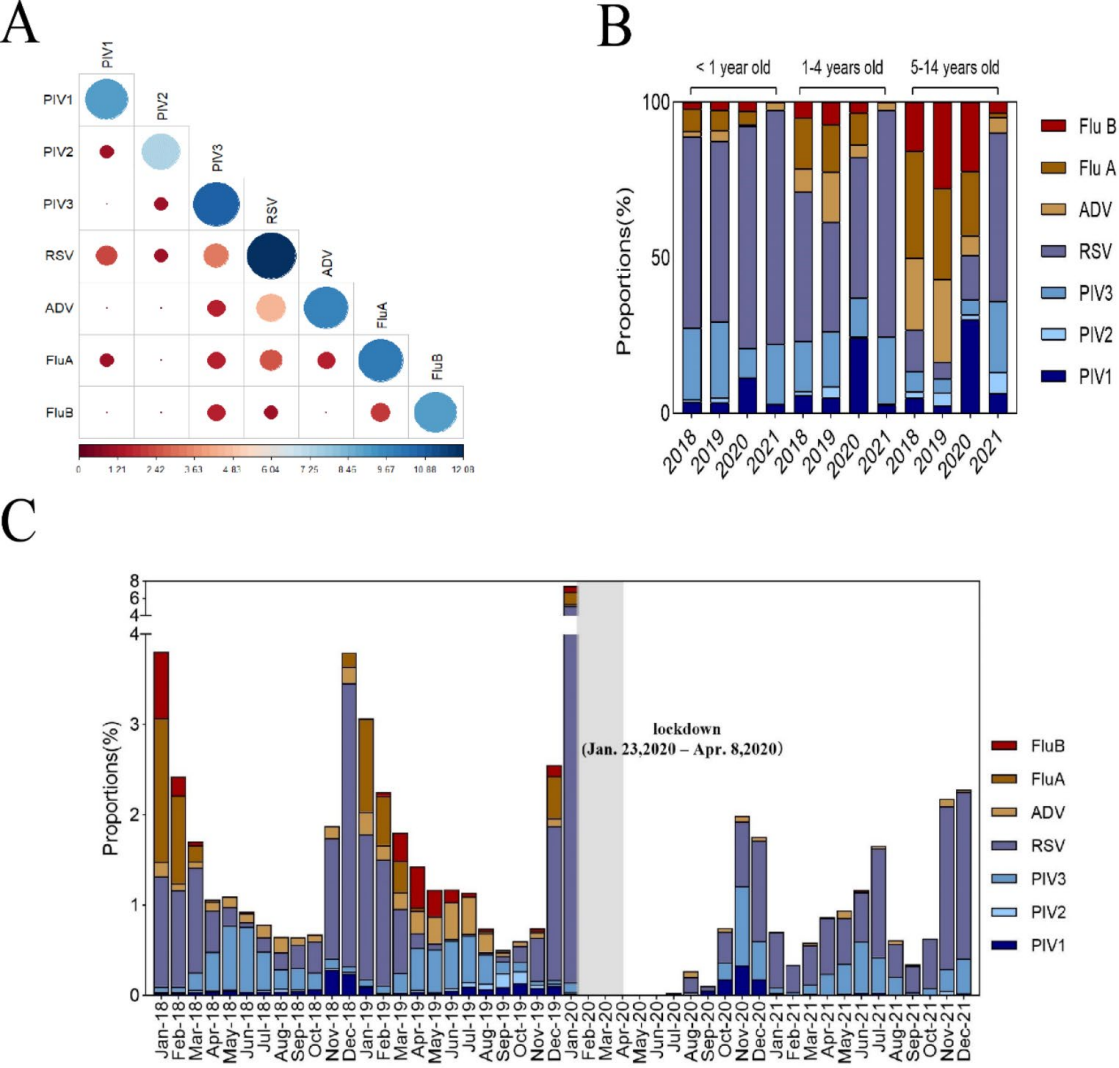
Coinfection and distribution data. (**A**) Viral combinations in coinfections. The horizontal axis represents log2 (Patient + 1); the darker the blue and the larger the dot, the larger the number of cases; the darker the red, the smaller the number of cases. (**B**) Frequency of detected viral pathogens in children by age group (2018–2021). (**C**) Monthly distribution of positive cases (2018–2021)

In total, 16.83% (9115/54171) of the children with an ARI tested positive for at least one pathogen. Compared with 2018 (3520/18035, 19.52%) and 2019 (3662/21471, 17.06%), the positive rate was significantly lower in 2021 (1238/9905, 12.50%) (Table 2). Among the single infections, RSV (4342/9115, 47.64%) was the most common, followed by PIV3 (1595/9115, 17.50%), influenza A virus (1133/9115, 12.43%), ADV (823/9115, 9.03%), influenza B virus (536/9115, 5.88%), PIV1 (520/9115, 5.70%), and PIV2 (166/9115, 1.82%) (Supplementary Table S2). The positive rate did not differ between boys and girls (10.07% *vs*. 6.76%, *P* = 0.386); however, it was significantly higher in the 1- to 4-year-old group than in the < 1 and 5- to 14-year-old groups (*P* = 0.098, Table 2). The rate of coinfection with two viruses was 0.79% (43/54 171); RSV was the most frequently occurring virus in coinfection cases (Fig. 3A).

**Table 2:** Demographics of positive cases

Category	2018 (n = 18,035)		2019 (n = 21,471)		2020 (n = 4760)		2021 (n = 9905)		Total (n = 54,171)		*P*
Boys	2109	11.69%	2196	10.23%	430	9.03%	720	7.27%	5455	10.07%	0.778
Girls	1411	7.82%	1466	6.83%	265	5.57%	518	5.23%	3660	6.76%	0.626
Age											
< 1 year	1075	5.96%	1043	4.86%	209	4.39%	274	2.77%	2601	4.80%	0.592
1–4 years	2127	11.79%	2105	9.38%	423	8.89%	903	9.12%	5558	10.26%	0.777
5–14 years	318	1.76%	514	2.39%	63	1.32%	61	0.62%	956	1.76%	0.816
Total	3520	19.52%	3662	17.06%	695	14.60%	1238	12.50%	9115	16.83%	

There was no significant difference in the number of patients with ARIs among the three age groups (*P* = 0.098)

In total, 9115 children tested positive for at least one viral pathogen. RSV was the most prevalent pathogen in children < 1 year and 1–4 years old (2018, 2019, and 2021). Influenza virus was the most frequently occurring virus in 2018 and 2019, but it was hardly detected at all in 2021 in the 5- to 14-year-old group. After the COVID-19 pandemic, RSV was the main pathogen involved in ARIs in the 5- to 14-year-old group (Fig. 3B, Supplementary Table S2).

### Monthly distributions

In this study, the positive detection rate started increasing in October and reached a peak in January of the following year (Fig. 3C). RSV detection peaked in January 2020 and showed a higher rate in 2021, after confinement, compared to the other six viruses. The detection rate of influenza viruses was significantly lower in 2021 than in 2018 and 2019. The detection rates of PIV1, PIV2, and ADV saw a large decline in 2021; for PIV3, the peak detection rate was reached in November 2020 (Fig. 3C, Supplementary Table S3).

## Discussion

This report describes the epidemiology of common ARIs in children under 14 years of age in Wuhan over a period of four years (2018–2021). We found that the number of patients with ARI and the positive detection rate decreased dramatically after the start of the COVID-19 pandemic. The population in Wuhan was confined from 23 January to 8 April 2020; however, the NPIs – including patient isolation, social distancing, hand washing, and mask wearing – are still ongoing during the COVID-19 pandemic. Many studies have explored the impact of NPIs on non-SARS-CoV-2 infections [24–27]. After the efforts and unprecedented NPIs put in place around the world, epidemic curves started to level off, and also due to good weather, COVID-19 hospitals started to empty, deaths rapidly decreased, and the number of cases showed a clear downward trend. However, most studies have not considered the period following the relaxation of NPIs. By comparing the number of patients with ARIs and the positive detection rate in 2021 with those in 2018 and 2019, we found a significant effect of NPIs; PIV, RSV, ADV, and influenza virus were frequently identified in children with ARIs. Nevertheless, the overall virus detection rate in 2021 was lower than that in 2018 and 2019. Similarly, a study at Children’s Hospital of Zhejiang University showed a decline of 59.9% and 57.4% in outpatient visits during the period of February-April 2020 as compared with the same periods in 2018 and 2019, respectively, and a decline of 65.7% and 59.0% in the total number of respiratory tract infections from January to April 2020 compared with the same periods in 2018 and 2019, respectively [28]. In Children’s Hospital of Fudan University, there was a decline in positive rates of most viruses, with the largest decrease observed for influenza A virus (-0.94%), followed by ADV, rotaviruses, and influenza B virus. However, the positive rates of RSV and enteric ADV rose during the post-COVID-19 period as the NPIs were relaxed [29]. To investigate the cause of the observed reduction in paediatric visits, a study in the Netherlands showed that a larger reduction was observed for communicable infections (76%) than for non-infectious diseases (36%), which means that the main reason for the reduction in paediatric visits was a decrease in transmissible infections due to the adoption of NPIs, and care avoidance could also have contributed [30]. In a study from the USA, influenza activity was found to be lower than during any previous influenza season, and other common viruses such as RSV and PIV did not show a similar epidemiological pattern [31].

Our study showed that younger children were more susceptible to viral infection than older children, which is consistent with findings from other studies [2–5]. By comparing the spectrum of pathogens between different age groups, we identified RSV as the leading pathogen in children aged < 1 year and 1–4 years, which is consistent with reports from other countries [5, 32]. It has been reported that the respiratory virus testing standards during the COVID-19 pandemic were lowered, which might have contributed to the observed increase in RSV-related disease and a change in age distribution [33]. The number of children visiting medical facilities decreased greatly during and after COVID-19, but the rate of infection with RSV was increasing [33]. Influenza virus was identified as the leading viral pathogen in 2018 and 2019 but declined dramatically in the post-COVID-19 period in children aged 5–14 years. Reports from other countries have shown similar trends [34–36]. These results demonstrated that the current NPIs could be highly effective against influenza. The wearing of surgical face masks was reported to significantly reduce the detection of influenza virus RNA in respiratory droplets in Hong Kong, indicating that their use could prevent the transmission of the virus from symptomatic individuals [37]. This positive effect in the short term is welcome. However, the lack of immune stimulation due to reduced circulation of influenza virus and to the related administration of vaccine may have caused an “immunity debt”, which could have negative consequences when the pandemic is under control and NPIs are lifted [38]. Further studies are needed to better understand how the immunity debt affects the epidemiology of influenza.

Compared with those of other viruses, the infection rate of RSV showed an increase in children aged 5–14 years after the COVID-19 outbreak. The reason for this phenomenon remains unclear. One possibility is an interaction between RSV and influenza virus. When multiple pathogens cocirculate, this can lead to competitive or cooperative forms of pathogen-pathogen interaction [38, 39]. A localized inflammatory response can be induced by infection with influenza virus that can restrict the replication of RSV [23, 40, 41]. Another possible reason is that the preventive measures taken against COVID-19 have changed the RSV spectrum. A recent study of the variation of RSV showed that RSV-B was predominant in 2020–2021 in Wuhan [42], while surveillance of RSV in China previously showed that RSV-A was dominant in most years [43]. In Tokyo, RSV activity declined by 97.9%, and the transmission rate decreased by 40% during the period when NPIs were enforced. Moreover, it was predicted using a regression model that a longer period of NPIs and a larger reduction in transmission rate may increase the susceptible population and subsequently lead to a larger outbreak in Japan [44]. Therefore, RSV could be the predominant respiratory virus, and evolution of the RSV genome should be monitored more diligently in children in the future.

Coinfection with multiple viruses can occur in children with ARIs. In our study, the coinfection rate was 0.84%, and RSV was the most prevalent pathogen involved in coinfections. This finding, together with that of RSV being the leading viral pathogen in children aged < 5 years, is consistent with the results of studies from China and other countries conducted before and after the COVID-19 pandemic [5, 24]. Therefore, prevention strategies for RSV are always essential.

We also found that the change in seasonality of respiratory viruses in different age groups varied. We observed that the number of patients with ARI and the virus detection rate were higher in December and January than in other months. In Wuhan, it is rainy and cold from November to January. Numerous studies have shown an association between the transmission of respiratory viruses and the climate, especially in terms of humidity and temperature [45–48].

It has been demonstrated that NPIs have contributed to reducing the transmission of SARS-CoV-2 [49–52], and the hospitalisation rate of children with ARI has decreased substantially [53–55]. After restrictions, especially confinement, were lifted, we observed a gradual resurgence of RSV and PIV3 infections. During the lockdown, children were less frequently exposed to pathogens than normal, which might have affected the development of “trained immunity”. The concept of trained immunity refers to long-term functional reprogramming of innate immune cells, stimulated by pathogens, which leads to a reinforced response during subsequent exposures. A weaker innate immune response might result in higher vulnerability to viruses in the post-COVID‐19 period. The longer these periods of low exposure to virus and bacteria last, the greater the likelihood of epidemics in the future. This is because there is a growing proportion of susceptible individuals and declining herd immunity in the population. The probable role of live vaccines administered during childhood in this trained immunity should be studied to balance these negative consequences.

Our study has several limitations. First, it is limited to a single region. The information might not be applicable to other regions. Second, we tested for the presence of seven specific respiratory viruses, and other viruses were not considered. Third, we used an immunofluorescence assay for virus detection rather than DNA sequencing. Immunofluorescence is inferior to the gold standard method, real-time PCR, but the large number of study participants might have compensated for the lack of sensitivity leading to false-negative results for patients with a low viral load. Lastly, apart from viral diagnosis, there was no further molecular or antigenic characterization of positive virus samples by methods such as sequencing and phylogenetic analysis to support the postulation of “immunity debt”.

## Conclusion

We analysed the epidemiological patterns of different respiratory viruses in paediatric patients at Renmin Hospital of Wuhan University (two districts) from 2018 to 2021. The COVID-19 pandemic has altered our daily behaviours, resulting in fewer patients with ARI, lower viral infection rates, and changes in the spectrum of pathogens in paediatric ARI cases. In Wuhan, the implemented NPIs resulted in fewer opportunities to spread common viruses but also brought potential dangers of increased susceptibility and decreased herd immunity in the population. Further studies are needed to clarify how the immunity debt affects the epidemiology of common respiratory viruses. Therefore, global surveillance of viral respiratory infections should continue over the long term.

### Electronic supplementary material

Below is the link to the electronic supplementary material.

Additional File 1: Supplementary Data.

## Data Availability

The datasets used and/or analyzed during the current study are available from the corresponding author on reasonable request.
