# Supplementary material for: The changing pattern of common respiratory viruses among children from 2018 to 2021 in Wuhan, China
Source: Arch Virol. 2023 Nov 14;168(12):291. doi: 10.1007/s00705-023-05891-7 (PMC10645662; doi:10.1007/s00705-023-05891-7)
Supplement: Supplementary file 1 — Additional File 1: Supplementary Data. [file 705_2023_5891_MOESM1_ESM.doc]

**Supplementary Data**

Supplementary Table 1. Distribution of monthly patient visits from 2018 to 2021

Supplementary Table 2. Distribution of viral pathogens among age groups

Supplementary Table 3. Monthly distribution of positive cases

**Supplementary Table 1 Distribution of monthly patient visits from 2018 to** 2021

| **Month** | **2018** | **2019** | **2020** | **2021** | **Total** |
| --- | --- | --- | --- | --- | --- |
|  | **n=18 035, No (%)** | **n=21 471, No (%)** | **n=4760, No (%)** | **n=9905, No (%)** | **n=51 471, No (%)** |
| **January** | 1713 (9.36) | 2028 (9.45) | 1240 (26.05) | 683 (6.90) | 5664 (11.00) |
| **February** | 1081 (5.91) | 1388 (6.46) | 0 (0.00) | 241 (2.43) | 2710 (5.27) |
| **March** | 1325 (7.24) | 1810 (8.43) | 0 (0.00) | 609 (6.15) | 3744 (7.27) |
| **April** | 1410 (7.70) | 1870 (8.71) | 10 (0.21) | 894 (9.03) | 4184 (8.13) |
| **May** | 1718 (9.39) | 1842 (8.58) | 28 (0.59) | 1061 (10.71) | 4649 (9.03) |
| **June** | 1644 (8.98) | 1830 (8.52) | 125 (2.63) | 1065 (10.75) | 4664 (9.06) |
| **July** | 1613 (8.81) | 1956 (9.11) | 246 (5.17) | 1036 (10.46) | 4851 (9.42) |
| **August** | 1131 (6.18) | 1605 (7.48) | 346 (7.27) | 457 (4.61) | 3539 (6.88) |
| **September** | 1303 (7.12) | 1713 (7.98) | 545 (11.45) | 534 (5.39) | 4095 (7.96) |
| **October** | 1605 (8.77) | 1720 (8.01) | 638 (13.40) | 723 (7.30) | 4686 (9.10) |
| **November** | 1629 (8.90) | 1745 (8.13) | 751 (15.78) | 1238 (12.50) | 5363 (10.42) |
| **December** | 1863 (10.18) | 1964 (9.15) | 831 (17.46) | 1364 (13.77) | 6022 (11.70) |

This table shows the number and proportions of monthly patient visits.

**Supplementary Table 2 Distribution of viral pathogens among age groups**

| **Positive cases** | | **PIV1** | | **PIV2** | | **PIV3** | | **RSV** | | **ADV** | | **Influenza A** | | **Influenza B** | |
| --- | --- | --- | --- | --- | --- | --- | --- | --- | --- | --- | --- | --- | --- | --- | --- |
| **2018**  **(n=3520)** | **<1 (n=1075)** | 39 | (3.63%) | 9 | (0.84%) | 247 | (22.98%) | 662 | (61.58%) | 18 | (1.67%) | 77 | (7.16%) | 23 | (2.14%) |
|  | **1–4 (n=2127)** | 124 | (5.83%) | 28 | (1.32%) | 342 | (16.08%) | 1019 | (47.91%) | 161 | (7.57%) | 346 | (16.27%) | 107 | (5.03%) |
|  | **5–14 (n=318)** | 16 | (5.03%) | 6 | (1.89%) | 21 | (6.60%) | 42 | (13.21%) | 74 | (23.27%) | 109 | (34.28%) | 50 | (15.72%) |
| **2019**  **(n=3662)** | **<1 (n=1043)** | 36 | (3.45%) | 18 | (1.73%) | 254 | (24.35%) | 605 | (58.01%) | 37 | (3.55%) | 66 | (6.33%) | 27 | (2.59%) |
|  | **1–4 (n=2105)** | 109 | (5.18%) | 75 | (3.56%) | 370 | (17.58%) | 740 | (35.15%) | 342 | (16.25%) | 318 | (15.11%) | 151 | (7.17%) |
|  | **5–14 (n=514)** | 13 | (2.53%) | 21 | (4.09%) | 23 | (4.47%) | 28 | (5.45%) | 137 | (26.65%) | 150 | (29.18%) | 142 | (27.63%) |
| **2020**  **(n=695)** | **<1 (n=209)** | 24 | (11.48%) | 0 | (0.00%) | 20 | (9.57%) | 149 | (71.29%) | 1 | (0.48%) | 9 | (4.31%) | 6 | (2.87%) |
|  | **1–4 (n=423)** | 103 | (24.35%) | 1 | (0.24%) | 53 | (12.53%) | 191 | (45.15%) | 17 | (4.02%) | 44 | (10.40%) | 14 | (3.31%) |
|  | **5–14 (n=63)** | 19 | (30.16%) | 1 | (1.59%) | 3 | (4.76%) | 9 | (14.29%) | 4 | (6.35%) | 13 | (20.63%) | 14 | (22.22%) |
| **2021**  **(n=1238)** | **<1 (n=274)** | 8 | (2.92%) | 0 | (0.00%) | 53 | (19.34%) | 206 | (75.18%) | 7 | (2.55%) | 0 | (0.00%) | 0 | (0.00%) |
|  | **1–4 (n=903)** | 25 | (2.77%) | 3 | (0.33%) | 195 | (21.59%) | 658 | (72.87%) | 22 | (2.44%) | 0 | (0.00%) | 0 | (0.00%) |
|  | **5–14 (n=61)** | 4 | (6.56%) | 4 | (6.56%) | 14 | (22.95%) | 33 | (54.10%) | 3 | (4.92%) | 1 | (1.64%) | 2 | (3.28%) |
| **Total** | **(n=9115)** | 520 (7.70) | | 166 (1.82) | | 1595 (17.50) | | 4342 (47.64) | | 823 (9.03) | | 1133 (12.43) | | 536 (5.88) | |

This tables shows the number and constituent ratio of viral pathogens among the different age groups.

**Supplementary Table 3 Monthly distribution of positive cases of ARI**

| **Detected cases** | **2018 (n=18 035)** | | **2019 (n=21 471)** | | **2020 (n=4760)** | | **2021 (n=9905)** | |
| --- | --- | --- | --- | --- | --- | --- | --- | --- |
| **Month** | **n=3520**  **(19.52%)** | | **n=3662**  **(17.06%)** | | **n=695**  **(14.60%)** | | **n=1238**  **(17.71%)** | |
| **January** | 688 | (3.81%) | 656 | (3.06%) | 357 | (7.50%) | 72 | (0.73%) |
| **February** | 438 | (2.43%) | 483 | (2.25%) | 0 | (0.00%) | 35 | (0.35%) |
| **March** | 309 | (1.71%) | 386 | (1.80%) | 0 | (0.00%) | 60 | (0.61%) |
| **April** | 192 | (1.06%) | 306 | (1.43%) | 0 | (0.00%) | 87 | (0.88%) |
| **May** | 199 | (1.10%) | 250 | (1.16%) | 0 | (0.00%) | 95 | (0.96%) |
| **June** | 168 | (0.93%) | 250 | (1.16%) | 0 | (0.00%) | 119 | (1.20%) |
| **July** | 143 | (0.79%) | 241 | (1.12%) | 3 | (0.06%) | 167 | (1.69%) |
| **August** | 118 | (0.65%) | 157 | (0.73%) | 15 | (0.32%) | 62 | (0.63%) |
| **September** | 117 | (0.65%) | 106 | (0.49%) | 13 | (0.27%) | 35 | (0.35%) |
| **October** | 122 | (0.68%) | 125 | (0.58%) | 60 | (1.26%) | 63 | (0.64%) |
| **November** | 340 | (1.89%) | 157 | (0.73%) | 139 | (2.92%) | 217 | (2.19%) |
| **December** | 686 | (3.80%) | 545 | (2.54%) | 108 | (2.27%) | 226 | (2.28%) |

This table shows the ARI monthly distribution and positive detection rate.
